# National action plan for non-communicable diseases prevention and control in Iran; a response to emerging epidemic

**DOI:** 10.1186/s40200-017-0288-4

**Published:** 2017-01-23

**Authors:** Niloofar Peykari, Hassan Hashemi, Rasoul Dinarvand, Mohammad Haji-Aghajani, Reza Malekzadeh, Ali Sadrolsadat, Ali Akbar Sayyari, Mohsen Asadi-lari, Alireza Delavari, Farshad Farzadfar, Aliakbar Haghdoost, Ramin Heshmat, Hamidreza Jamshidi, Naser Kalantari, Ahmad Koosha, Amirhossein Takian, Bagher Larijani

**Affiliations:** 1 Iranian Non Communicable Diseases Committee (INCDC), Ministry of Health and Medical Education, Tehran, Iran; 2 INCDC, Ministry of Health and Medical Education, Tehran, Iran; 3 Food and Drug Organization, INCDC, Ministry of Health and Medical Education, Tehran, Iran; 4 Deputy of Curative Affairs , INCDC, Ministry of Health and Medical Education, Tehran, Iran; 5 Deputy of Research and Technology, INCDC, Ministry of Health and Medical Education, Tehran, Iran; 6 Deputy of Development, Management, and Resources, INCDC, Ministry of Health and Medical Education, Tehran, Iran; 7 Deputy of Public Health, INCDC, Ministry of Health and Medical Education, Tehran, Iran; 8 International Affairs, INCDC, Ministry of Health and Medical Education, Tehran, Iran; 9 Digestive Disease Research Center, Tehran University of Medical Sciences, and INCDC, MOHME, Tehran, Iran; 10 Non-Communicable Diseases Research Center, EMRI, Tehran University of Medical Sciences, Tehran, Iran; 11 Kerman University of Medical Sciences, Kerman, Iran; 12 Chronic Diseases Research Center, EMRI, Tehran University of Medical Sciences, Tehran, Iran; 13 Shahid Beheshti University of Medical Sciences, Tehran, Iran; 14 Tabriz University of Medical Sciences, Tabriz, Iran; 15 Center for NCDs control and prevention, and INCDC, MOHME, Tehran, Iran; 16 Tehran University of Medical Sciences, Tehran, Iran; 17 INCDC, Ministry of Health and Medical Education, and EMRI, TUMS, Tehran, Iran

**Keywords:** Non-communicable diseases, Action plan, Iran

## Abstract

Emerging Non-communicable diseases burden move United Nation to call for 25% reduction by 2025 in premature mortality from non-communicable diseases (NCDs). The World Health Organization (WHO) developed global action plan for prevention and control NCDs, but the countries’ contexts, priorities, and health care system might be different. Therefore, WHO expects from countries to meet national commitments to achieve the 25 by 25 goal through adapted targets and action plan.

In this regards, sustainable high-level political statement plays a key role in rules and regulation support, and multi-sectoral collaborations to NCDs’ prevention and control by considering the sustainable development goals and universal health coverage factors.

Therefore, Iran established the national authority’s structure as Iranian Non Communicable Diseases Committee (INCDC) and developed NCDs’ national action plan through multi-sectoral approach and collaboration researchers and policy makers. Translation Iran’s expertise could be benefit to mobilizing leadership in other countries for practical action to save the millions of peoples.

## Background

Alarming increase of premature mortality from non-communicable diseases across the world considered by Global action plan on NCDs and Sustainable Development Goals [1–3]. Also, the High-Level Meeting on NCDs in the United Nations emphasized on the need of all government policy response to this dramatic problem [4]. NCDs were responsible to more than 38 million deaths in the world and annually, non-communicable diseases responsible for 16 million premature death [5]. This staggering toll of non-communicable diseases and premature mortality leads policy makers to pay attention and instigate action across the globe [6].

Evidences indicate that, Iran threatened by NCDs too [7–10]. In Iran, during the recent decades, the socio-economic development and the successful function of Primary Health Care (PHC) bring out health promotion, child, and maternal mortality decrease [11]. In addition, life expectancy increased from 66 to 78 years in period of 1990 to 2013.

Following these progresses in health situation of Iranians, a remarkable revolution occurred in the health status of the country especially in the field of communicable diseases. Nevertheless, non-communicable diseases remained as the great health problem in Iran. In 2013, 236 thousand deaths in Iran occurred due to NCDs and there was 14.5% increase of NCDs’ death during two past decades [12]. At the same time, mental disorders, substance abuse and traffic injuries were cause of 15821 deaths. It is remarkable, 82.2% of NCDs’ death is due to cardiovascular diseases, cancers, chronic pulmonary diseases, and diabetes and these are top ranks of cause of death in the country [12]. In Iran, dietary risks are the first line of NCDs’ risk factors and metabolic risk factors stand at second position. Tobacco smoke, air pollution, low physical activity, and alcohol and drug use are the next related risk factors, respectively [12].

In 2013, NCDs led to 7 million years lived with disability (YLD) in Iran [13]. It is considerable, NCDs accompanied with economical burden because of productivity reduction and diverting resources from productive purposes to NCDs treatment. Because of, the poor population struggled with health expenditure and living costs, they have been suffered from economic stress more than other people [12, 14]. It is remarkable; the first cause of catastrophic health expenditures is NCDs and this problem reinforce societal inequities [6, 15].

Non-communicable diseases ignored during the framing of the Millennium Development Goals in 2000, but clarification of the reality leads the policymakers to pay attention to it. The United Nations and the World Health Organization (WHO) have called for a 25% reduction by 2025 in mortality from non-communicable diseases among persons between 30 and 70 years of age, in comparison with mortality in 2010, adopting the slogan “25 by 25” [4].

Universal health coverage (UHC) as a critical component of sustainable development reduces poverty and social inequities through financial risk protection. It is important; the Key elements of the right to health are availability, accessibility, acceptability, quality [16].

By considering these rights and responding to significant burden of NCDs, the World Health Organization (WHO) developed the global action plan for the prevention and control of NCDs (2013–2020) which contain 9 targets by considering four main risk factors; including tobacco use, an unhealthy diet, lack of physical activity and harmful alcohol use. These behavioral risk factors are associated with four main disease clusters mentioned above [17]. As many NCDs’ death and disability are preventable by appropriate interventions, this action plan provides policy options for countries to strengthen them to reduce premature deaths from NCDs by 25% by 2025 [18].

The WHO has messaged; the countries could shift from political commitment to effective action by prioritizing affordable interventions. This organization emphasized on the need of all countries to set national NCDs targets and responsible to attain them [19, 20].

The aim of this paper is to present the emerging Iranian architecture for NCDs’ prevention and control and create NCDs’ national action plan to move forward.

## Response to emerging NCDs’ epidemic

### Political commitment

Mandate with the task of developing a national action plan for NCDs’ prevention and control was the opportunity for Iran to commitment 25 by 25 NCDs goal [21]. In context of national action plans, we could arrange feasible, scalable, affordable, and cost effective interventions to reduce NCDs’ mortality and morbidity and attain to the most efficient and equitable health system [22, 23].

Success in response to NCDs crisis depends on strong and effective leadership in high-level political statement [24]. In this way, health policies support regulatory, legislative and inter-sectoral collaborations to address equity and reduction NCDs’ outcomes.

### Establishment sustainable national leadership

Ministry of Health and Medical Education (MOHME) of Iran as strong and effective leadership in high-level political statement established Iranian non-communicable diseases committee (INCDC) with the aim of comprehensive policy making, planning, and monitoring of all activities in the area of non-communicable diseases in Iran.

**Fig. 1:**
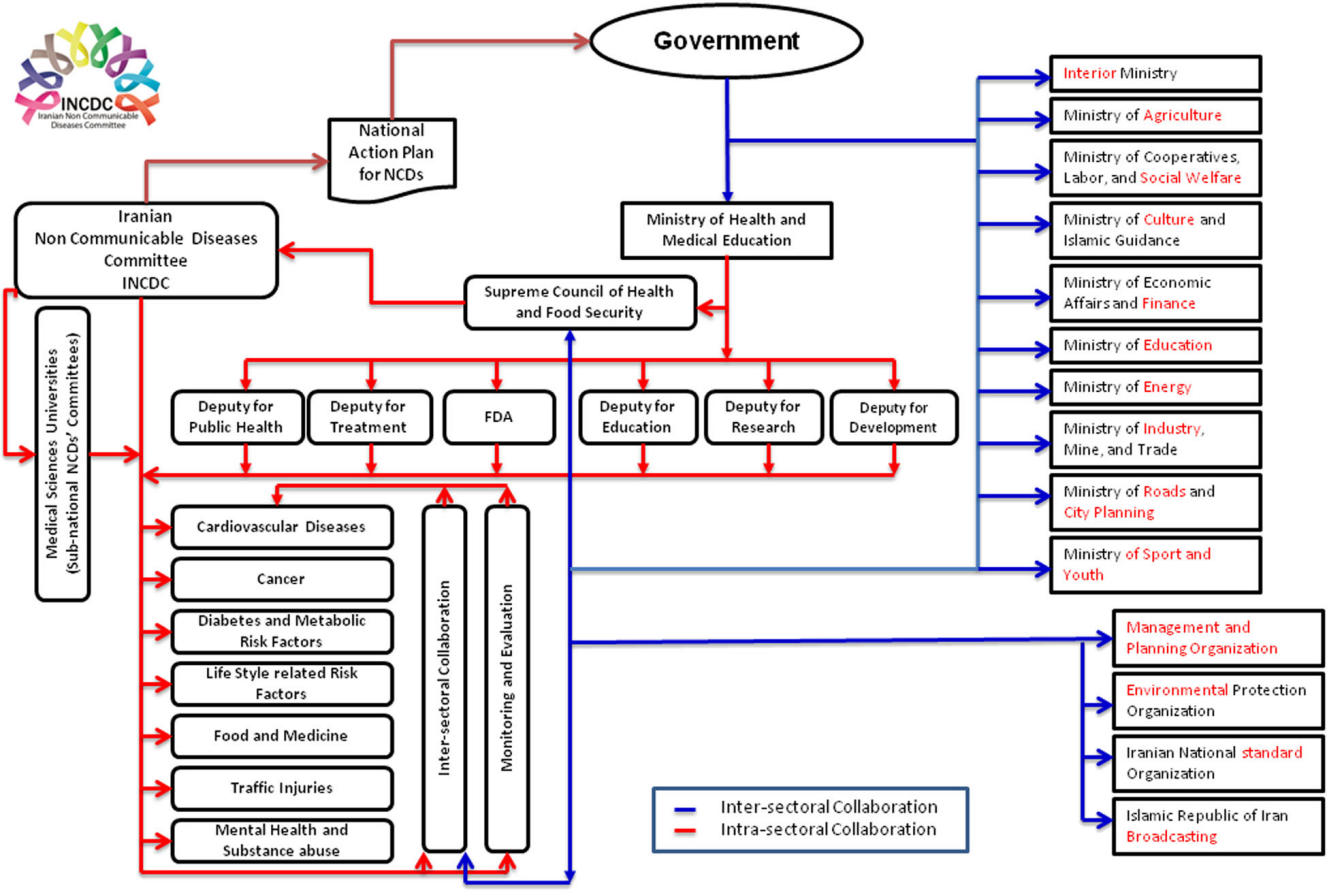
The inter and intra- sectoral collaboration of Iranian Non Communicable Diseases Committee

As shown in Fig. 1, INCDC set up by inter and intra-sectoral collaboration. The presented flow demonstrates the relationships between inter and intra-sectoral parts. The body of INCDC contains ministry of health deputies, supreme council of health and food security and related to medical sciences universities.

Iranian non-communicable diseases committee has nine sub-committees according to various aspects of prevention and controls NCDs and their risk factors and national targets (Fig. 1).

### A stepwise move toward action

#### First step: setting clear and appropriate targets in national level

Planning is one of the key steps for responding to NCDs’ dramatic epidemic [25]. “*National Action Plan for Prevention and Control of Non-Communicable Diseases and the related risk factors in Iran, 2015-2025”* was developed by considering our country’s priorities, National and Sub-national Burden of Diseases, and WHO global targets [1, 26–29].

The national action plan for NCDs Prevention and Control in Iran including 9 targets based on global action plan for NCDs and 4 special targets regarding trans fatty acid, traffic injuries, drug abuse, and mental diseases.

Among nine global targets on prevention and control NCDs, target 3 and target 8 adapted for our country situation. According to WHO report, Prevalence of insufficient physical activity in Iranian adult was 24.1% among men and 42.9% among women [30, 31]. The 3^th^ target of NCDs global action plan set to reduce physical inactivity by 10% by 2025. According to our country situation, we considered 20% reduction of physical inactivity.

In addition, the eighth target of global action plan set to receiving drug therapy and counseling to prevent heart attacks and strokes by 50% of eligible people. But we accelerate this desired measure to 70% because of access to medicines plays a vital role in universal health coverage goals achievement and already we stand up good point [32]. It is remarkable, the other targets were appropriate for Iran by considering our conditions and situation analysis.

In addition to glossary WHO targets, United Nation proposed Sustainable Development Goals (SDGs). The third sustainable development goal focused on health and considered UHC as a mean of health equity. It is needed to effective attention to non-communicable diseases [33].

By considering burden of diseases in Iran, targets 10 to 13 set for our country (Table 1).

**Table 1:** Iranian National Action Plan Targets on NCDs and Sustainable Development Goals (SDGs)

Iranian National Action Plan Targets on NCDs	Sustainable Development Goals	Universal Health Coverage Goals	Health System Goals
Target 1.25% relative reduction in the risk of premature death from cardiovascular disease, cancer, diabetes, chronic respiratory diseases	*SDGs Target 3.4*.By 2030, reduce by one third premature mortality from non-communicable diseases through prevention and treatment and promote mental health and well-being	Utilization Quality Financial protection	Responsiveness Health gain and equity in health Financial protection and equitable finance
Target 2. At least 10% relative reduction in alcohol consumption	*SDGs Target 3.5.* Strengthen the prevention and treatment of substance abuse, including narcotic drug abuse and harmful use of alcohol		
Target 3. A 20% relative reduction in prevalence of insufficient physical activity	*SDGs Target 3.d.*Strengthen the capacity of all countries, in particular developing countries, for early warning, risk reduction and management of national and global health risks		
Target 4.30% relative reduction in the average salt intake in the population	*SDGs Target 3.d.* Strengthen the capacity of all countries, in particular developing countries, for early warning, risk reduction and management of national and global health risks		
Target 5.30% relative reduction in the prevalence of tobacco use in persons aged 15+ years	*SDGs Target 3a.* Strengthen the implementation of the World Health Organization Framework Convention on Tobacco Control in all countries, as appropriate		
	*SDGs Target 3.d.* Strengthen the capacity of all countries, in particular developing countries, for early warning, risk reduction and management of national and global health risks		
Target 6.25% relative reduction in the prevalence of high blood pressure or contain the prevalence of raised blood pressure	*SDGs Target 3.d.* Strengthen the capacity of all countries, in particular developing countries, for early warning, risk reduction and management of national and global health risks		
Target 7. Halt the rates of diabetes and obesity	*SDGs Target 3.d.* Strengthen the capacity of all countries, in particular developing countries, for early warning, risk reduction and management of national and global health risks		
Target 8. At least 70% of eligible people receive drug therapy and counseling to prevent heart attacks and strokes	*SDGs Target 3.8.* Achieve universal health coverage, including financial risk protection, access to quality essential health-care services and access to safe, effective, quality and affordable essential medicines and vaccines for all		
Target 9. An 80% availability of the affordable basic technologies and essential medicines, including generics in private and public sectors	*SDGs Target 3.8.* Achieve universal health coverage, including financial risk protection, access to quality essential health-care services and access to safe, effective, quality and affordable essential medicines and vaccines for all		
	*SDGs Target 3b.* Support the research and development of vaccines and medicines for the communicable and non-communicable diseases that primarily affect developing countries, provide access to affordable essential medicines and vaccines, in accordance with the Doha Declaration on the TRIPS Agreement and Public Health, which affirms the right of developing countries to use to the full the provisions in the Agreement on Trade-Related Aspects of Intellectual Property Rights regarding flexibilities to protect public health, and, in particular, provide access to medicines for all		
	*SDGs Target 3.c.* Substantially increase health financing and the recruitment, development, training and retention of the health workforce in developing countries, especially in least developed countries and small island developing States		
Target 10. Zero Trans fatty acid in food & oily products	*SDGs Target 3.d.* Strengthen the capacity of all countries, in particular developing countries, for early warning, risk reduction and management of national and global health risks		
Target 11.20% Relative reduction in mortality rate due to traffic injuries	*SDGs Target 3.6.*By 2020, halve the number of global deaths and injuries from road traffic accidents		
Target 12. A 10% relative reduction in mortality rate due to drug abuse	*SDGs Target 3.5.* Strengthen the prevention and treatment of substance abuse, including narcotic drug abuse and harmful use of alcohol		
Target 13.20% increase in access to treatment for mental diseases	*SDGs Target 3.4*.By 2030, reduce by one third premature mortality from non-communicable diseases through prevention and treatment and promote mental health and well-being		

In Table 1, 13 national targets of Iran and their relationship with Sustainable Development Goals presented [2, 34–36]. UHC and health system goals are related to all NCDs targets and all of them are in the way of poverty reduction.

#### Second step: mobilization, inter & intra-sectoral collaboration and developing national action plan for NCDs prevention and control in Iran

In developing NCDs’ national action plan of Iran, inter and intra-sectoral collaboration was considered. INCDC initiated dialogue with health system sectors and the other ministries to response NCDs’ prevention and control. This multi-sectoral procedure and collaborating approach led to develop the national document based on recommended WHO framework contain four areas of governance, prevention and reduction of risk factors, health care, and surveillance, monitoring and evaluation.

Through collaborative planning, the time bounded national action plan was prepared based on clear policies with clarified targets, strategies and activities and determined stakeholders, desired outcome, evaluation criteria, and resources [34].

#### Third step: enlist the support of national and international policy makers

Prepared NCDs’ action plan supported by President, parliament speaker, Vice President, Head of Management and Planning Organization, Head of Environmental Protection Organization, Head of Islamic Republic of Iran Broadcasting, and nine Ministers in the government Cabinet. This holistic political support and responsibility might be unique in the region. It is remarkable, supreme council of health, chaired by president approved “National Action Plan for Non-Communicable Diseases Prevention and Control in Iran” and minister of health directed medical sciences universities to implement it in order to health promotion of community as the main stakeholder through sub-national NCDs’ committee.

#### Forth step: participation of sub-national policy makers

Iran has 61 Medical Sciences University which their health services covered all provinces of Iran. Integration of medical education and health in the Ministry of Health and Medical Education create appropriate atmosphere for strengthening the health care system in Iran [37, 38]. Towards realizing NCDs’ prevention and control targets, INCDC invited Medical Sciences Universities’ chancellors as the main authorities in sub-national health sectors. They could be directed provincial committees through collaborative arrangements to address local priorities and should be committed to multi-sectoral approach. This approach is essential among planning, implementation and accountability as key stages of response to NCDs’ problem [25].

To accelerate country response to non-communicable diseases prevention and control, policy makers could follow these strategies to strengthen governance, risk reduction, health care, and surveillance. Generating national and sub-national information and monitor the trends of NCDs and their risk factors, establishing health promotion programs across the life course through primary health care system and universal health coverage components, and tackling issues through multi-sectoral approach are critical strategies be suggested.

## Conclusion

The structured and stepwise approach of Iran towards NCDs prevention and control bring out a proposed way in the mixture of challenges and successes. Our experiences indicate that the Strong leadership needed to pass the way of fighting against NCDs. We emphasized on this principle; the successful response to NCDs need to multi-sectoral approach, the engagement of all health sectors and related stakeholders including other ministries and organizations [34].

Iranian Non-communicable Diseases Committee (INCDC) is an appropriate model in supreme level for mega coordination [39, 40]. According to NCDs’ progress monitor report of WHO, Iran stand up the first rank in Eastern Mediterranean region and could be share lesson learned to other countries [41].

Resource mobilization, technical allocation, health workforce development and health care quality improvement are essential for action plan implementation. Poor leadership, change management, financial constraints, poor health infrastructure, inadequate qualified human resources, poor information and communication, poor coordination and resistance of some organizations might be lead to implementation collapse [42, 43]. But, clear vision and mission, stability of political factors, local authorities support, financial resources management, motivating private sector and emerging economic powers, Internal monitoring and evaluation, strengthening supportive supervision and increasing motivation through recognition of best practice could be benefit for successful implementation.

Overcoming possible barriers in action plan implementations, scaling up interventions and health management in various levels of health services leads to reduce years of life lost (YLL) and YLD due to NCDs and finally benefit for health equity and financial protection [44].

President, parliament, supreme council of health members and cabinet ministers’ support and collaboration with other ministries and organizations were unique experience in the region. Access to efficient epidemiological information, explicit views of national policy makers in various domains of public health, treatment, food and medicine, education, and research. Partnership of policy and decision makers, scientist, researchers and health experts in national action plan development, establishment national sub committees guided by national authorities and structuring the provincial committees as the frontline of health services were our strengths.

Of course, we faced to some challenges in resource mobilization. Despite our long-term goals, INCDC was not finance by specific and sufficient budget. In addition, lack of private health sector and Nongovernmental organizations’ collaboration was the other limitation could be considered in the way forward. The other important challenge was incompleteness and misclassification reporting of cause of death that addressed by National and Sub-national Burden of Diseases Study (NASBOD) and now, we have comprehensive atlas of death in by cause in various levels [26].

Finally, Similar to other experience, we suggest integration of NCDs’ prevention and control in primary health care, enforce appropriate interventions led by provincial committee, technical use of existing infrastructure and human resources, innovative resource mobilization such as funding through nongovernmental resource, provide progress monitoring system and ensure accountability from the involved sectors, and actions to accelerate progress [20, 36, 45].

## Abbreviations

INCDC: Iranian Non Communicable Diseases Committee
MOHME: Ministry of Health and Medical Education
NASBOD: National and Sub-national Burden of Diseases Study
NCDs: Non-communicable diseases
PHC: Primary health care
SDGs: Sustainable development goals
UHC: Universal health coverage
WHO: World Health Organization
YLD: Years lived with disability
YLL: Years of life lost
